# The Role of Machine Learning and Radiomics for Treatment Response Prediction in Idiopathic Normal Pressure Hydrocephalus

**DOI:** 10.7759/cureus.18497

**Published:** 2021-10-05

**Authors:** Houman Sotoudeh, Zahra Sadaatpour, Ali Rezaei, Omid Shafaat, Ehsan Sotoudeh, Mohsen Tabatabaie, Aparna Singhal, Manoj Tanwar

**Affiliations:** 1 Radiology, University of Alabama at Birmingham School of Medicine, Birmingham, USA; 2 The Russell H. Morgan Department of Radiology and Radiological Science, Johns Hopkins University School of Medicine, Baltimore, USA; 3 Surgery, Iranian Hospital in Dubai, Dubai, ARE; 4 Health Information Management, Arak University of Medical Sciences, Arak, IRN

**Keywords:** magnetic resonance imaging, artificial intelligence, radiomics, machine learning, normal pressure hydrocephalus

## Abstract

Introduction

Ventricular shunting remains the standard of care for patients with idiopathic normal pressure hydrocephalus (iNPH); however, not all patients benefit from the shunting. Prediction of response in advance can result in improved patient selection for ventricular shunting. This study aims to develop a machine learning predictive model for treatment response after shunt placement using the clinical and radiomics features.

Methods

In this retrospective pilot study, the medical records of iNPH patients who underwent ventricular shunting were evaluated. In each patient, the “idiopathic normal pressure hydrocephalus grading scale” (iNPHGS) and a “Modified Rankin Scale” were calculated before and after surgery. The subsequent treatment response was calculated as the difference between the iNPHGS scores before and after surgery. iNPHGS score reduction of two or more than two were considered as treatment response. The presurgical MRI scans were evaluated by radiologists, the ventricular systems were segmented on the T2-weighted images, and the radiomics features were extracted from the segmented ventricular system. Using Orange data mining open-source platform, different machine learning models were then developed based on the presurgical clinical features and the selected radiomics features to predict treatment response after shunt placement.

Results

After the implementation of the inclusion criteria, 78 patients were included in this study. One hundred twenty radiomics features were extracted, and the 12 best predictive radiomics features were selected. Using only clinical data (iNPHGS and Modified Rankin Scale), the random forest model achieved the best performance in treatment prediction with an area under the curve (AUC) of 0.71. Adding the Radiomics analysis to the clinical data improved the prediction performance, with the support vector machine (SVM) achieving the highest rank in treatment prediction with an AUC of 0.8. Adding age and sex to the analysis did not improve the prediction.

Conclusion

Using machine learning models for treatment response prediction in patients with iNPH is feasible with acceptable accuracy. Adding the Radiomics analysis to the clinical features can further improve the predictive performance. SVM is likely the best model for this task.

## Introduction

Normal pressure hydrocephalus (NPH) was first described in 1965 by Hakeem and Adams in patients with ventriculomegaly without elevated intracranial pressure [1]. Moreover, they reported clinical triad of urinary incontinence, dementia, and gait abnormality in these patients. Since then, the NPH has remained a diagnostic challenge. In addition to the classical triad, additional neurologic symptoms such as sexual dysfunction and psychiatric manifestation have been reported in NPH. Besides, there is a significant overlap between the clinical presentation of NPH and other neurodegenerative disorders such as Parkinson's disease and Alzheimer's dementia. NPH is a challenging diagnosis for neuroimaging community; even by utilizing advanced imaging modalities, differentiation between NPH and excavatum ventriculomegaly secondary to age-related cerebral volume loss is a daunting task [2]. It is believed that the prevalence of the NPH is about 1.3% in patients older than 65 years old [3], and up to 80% of patients with NPH remain undiagnosed [4]. NPH is divided into idiopathic (iNPH) and secondary types. In the idiopathic subtype, there is no prior history of the central nervous systems (CNS) disease; however, the secondary type is believed to be secondary to fibrosis and adhesions in the subarachnoid space secondary to previous CNS pathologies, such as meningitis, trauma, malignancy, and intracranial hemorrhages. Regardless of iNPH physiopathology, ventriculoperitoneal shunt placement to drain the CSF remains the standard of care in patients with iNPH for many decades [5].

It will make great clinical sense if physicians and researchers can predict the treatment response after shunting in these iNPH patients in advance. Several published studies have tried to explore this avenue. Currently, the positive “spinal tap test” (gait improvement after drainage of a large amount of CSF during lumbar puncture) is the main predictive test, but with an overall ‘flip-coin’ accuracy of about 53% [6-8]. It appears that the patient's age is not associated with treatment response [9]. Treatment response prediction by neuroimaging is also controversial. In several studies, the radiologist-driven characteristics, including disproportionately enlarged subarachnoid space hydrocephalus (DESH) sign, Evans-index, callosal angle (CA), and periventricular T2 FLAIR hyperintensities, were not associated with treatment response [10]. However, in several other studies, DESH sign, augmented post-ischemic CSF space, CA, and anterior callosal angle (ACA) were found to be associated with treatment response [11].

The lack of clear returns on conventional neuroimaging in predicting the treatment response can be partially attributed to the inability of the human eyes to detect numerous imaging features. A few radiologist-driven features such as DESH sign, Evans-index, CA, periventricular T2 FLAIR hyperintensities, and ACA probably do not contain enough predictive information. In this context, radiomics analysis has gained a strong research momentum and is expected to impact multiple areas of radiology. In radiomics, the regions of Interest/lesions are usually segmented by the radiologists, and then hundreds and thousands of the features are extracted from the segmented area by the software. These features are essentially the relationship between the different pixels/voxels within the segmented regions. Subsequently, the most predictive features will be selected and used to develop the machine learning models for different predictions (e.g., tumor grade, gene mutation in tumors, patient’s survival, treatment response, etc.). Radiomics was first developed mainly for oncologic imaging and benefits are well published. However, the application of radiomics is not limited to tumors, and this technique can be used to evaluate benign non-oncologic conditions as well [12].

This study aims to develop a machine learning model based on the clinical and neuroimaging data to predict the treatment response in iNPH after shunt placement. However, instead of radiologist-driven imaging features, we present the benefits of hybrid radiomics analysis.

## Materials and methods

This feasibility study was approved by the ethical committee of our university institutional review board (IRB). The IRB waived the requirement to obtain any additional informed consent. Medical records of patients with a diagnosis of iNPH were evaluated retrospectively from 2009 to 2021. Before medical chart review, the researchers (ZS, AR, and HS) were trained to extract the medical data from the patients' records based on the three variables of iNPH grading scale [13], as well as the Modified Rankin Scale to evaluate the patients’ performance (Table 1 and Table 2).

**Table 1:** Idiopathic normal pressure hydrocephalus (iNPH) grading scale (sum of A, B, and C).

	Findings	Scores
A. Cognitive impairment	Normal	0
	Complaints of amnesia or inattention, but no objective memory and attentional impairment	1
	Existence of amnesia or inattention, but no disorientation of time and place	2
	Existence of disorientation of time and place, but the conversation is possible	3
	Disorientation for the situation or meaningful conversation impossible	4
B. Gait disturbance	Normal	0
	Complaints of dizziness of drift and dysbasia, but no objective gait disturbance	1
	Unstable but independent gait	2
	Walking with any support	3
	Walking not possible	4
C. Urinary disturbance	Normal	0
	Pollakiuria or urinary urgency	1
	Occasional urinary incontinence (1–3 or more times per week, but less than once per day)	2
	Continuous urinary incontinence (1 or more times per day)	3
	Bladder function is almost or entirely deficient	4

**Table 2:** Modified Rankin Scale.

Findings	Scores
No symptoms	0
No significant disability. Able to carry out all usual activities, despite some symptoms	1
Slight disability. Able to look after own affairs without assistance, but unable to carry out all previous activities	2
Moderate disability. Requires some help but able to walk unassisted	3
Moderately severe disability. Unable to attend to own bodily needs without assistance and unable to walk unassisted	4
Severe disability. Requires constant nursing care and attention, bedridden, incontinent	5

The consecutive patients with iNPH who underwent ventriculoperitoneal shunt placement and had clinical follow-up after surgery were included in this study. Those with any other known CNS pathologies, including known neurodegenerative disorder other than NPH, history of brain tumors, old infarctions, encephalomalacia, prior surgery, prior CNS infections, and patients with incomplete follow-up after surgery were excluded. As commonly prevalent, white matter disease was not considered as an exclusion criteria, and patients with any grade of periventricular T2/FLAIR hyperintensities were included. Clinical presentations of the included patients were documented using a questionnaire based on the already described iNPH and the Modified Rankin Scale (ZS and AR). The most recent brain MRI before surgery was also collected for each patient. For each patient, after normalization of the images, the ventricular system, including the lateral, third, and fourth ventricles were manually segmented on T2-weighted sequence using the 3D slicer software package [14]. The segmentation was performed independently by two board-certified radiologists, each with 12 years of experience (ZS and AR), and the segmentation was confirmed by a third board-certified neuroradiologist with 13 years of experience (HS). All radiologists were blind to the post-surgical outcomes at the time of segmentation. Subsequently, different Radiomics features were extracted for each patient using the Pyradiomics library [15]. For treatment response assessment, the changes in the iNPH scale before and after surgery were used. The sum of 11 iNPH criteria was calculated for these two time points. The patients with two and more than two scale decrease were considered “responders.” The patients with one or less than one scale decrease were considered as “non-responders.”

The radiomics features were first normalized. Considering responder/non-responder status as the target end-points, the radiomics features were evaluated by Least Absolute Shrinkage and Selection Operator (Lasso) and “Information Gain Ratio” to select the most predictive features. Subsequently, different machine learning models, including logistic regression, decision tree, random forest, neural network, AdaBoost, k-nearest neighbors, and Support Vector Machine (SVM) models, were implemented using the clinical data (iNPH grading scale and Modified Rankin Scale) as well as selected radiomics features to predict responder versus non-responder patients after shunt placement. The performance of each model was tested using leave-one-out cross-validation technique. The performance of each model was reported based on the area under the curve (AUC), accuracy, sensitivity, and specificity. AUC was used to select the best model. The machine learning training testing was performed by the “Orange Data mining” platform [16]. The demographic data were analyzed by SPSS (Version 27.0. Armonk, NY: IBM Corp).

The detailed structure for each model includes KNN (Number of neighbors: 8, Metric: Euclidean, and Weight: Uniform), Decision Tree ( Induce binary tree, Minimum number of instances in leave: 9, No split subsets smaller than 8, Maximum tree depth: 100, and Stop when majority reaches: 95%), Random Forest (Number of trees: 30, Replicable training, No split subsets smaller than 9), SVM ( Cost: 1.7, Regression loss epsilon: 0.2, Kernel: Sigmoid, Optimization Numerical Tolerance: 0.01, and Optimization Iteration Limit: 100), Logistic Regression (Regularization type: Ridge L2, Strength C=1), AdaBoost (Base estimator: Tree, Number of estimators: 57, Learning rate: 1, Classification algorithm: SAMME.R, and Regression loss function: Linear), and Neural Network (Neurons in the hidden layers: 100,50,20, Activation: tanh, Solver: Adam, Regularization α=0.0001, and Maximum number of iterations: 300).

## Results

After reviewing the medical records, 111 patients diagnosed with iNPH underwent VP shunting in our center from 2009 to 2021. After the implementation of inclusion and exclusion criteria, 78 patients were included in the final analysis. The patients include 47 males and 31 females with an average age of 69.4 ± 9 years. The average iNPH grading scale was 4.19, and the average Modified Rankin Scale was 2.45. The average time between the surgery and follow-up was 7.3 weeks. After shunt placement, 34 patients (two or more than two score interval decrease) were considered as responders, and 44 were considered as non-responders (Table 3).

**Table 3:** Demographic characteristics of the patients. We found no statistical association between patients’ sex (*) and age (**) with the outcome.

Cases	Sex		P-value	Age		
				Mean	Standard Deviation	P-value
Responder	Female	12 (35.3 %)	0.49*	70	8.2	0.14**
	Male	22 (64.7 %)		71.7	9.1	
Non-responder	Female	19 (43.1 %)		69.8	9.7	
	Male	25 (56.9 %)		66.8	9	
Total		78		69.4	9	

For each patient, 120 radiomics features were extracted from the ventricular system on T2-weighted images. After implementation of the LASSO (alpha 0.01) and Information Gain Ratio, 12 Radiomics features were selected as the most predictive features to predict responder versus non-responder. The selected features include, DifferenceAverage, DifferenceEntropy, Contrast, ZonePercentage, SmallDependenceEmphasis, Skewness, LongRunEmphasis, RunVariance, Id, Idm, ZoneEntropy, and LargeAreaLowGrayLevelEmphasis.

Using four clinical features (1. Cognitive impairment, 2. Gait disturbance, 3. Urinary incontinence from the iNPH grading scale, and 4. Modified Rankin Scale), the random forest model achieved the best performance to predict responder/non-responder target with an AUC of 0.71 (Table 4).

**Table 4:** The performance of different machine learning models to predict the outcome using various clinical features. AUC: area under the curve; SVM: support vector machine.

Model	AUC	Accuracy	Sensitivity	Specificity
Decision tree	0.60	0.55	0.44	0.63
SVM	0.63	0.59	0.61	0.56
Neural network	0.66	0.67	0.61	0.72
Logistic regression	0.70	0.64	0.58	0.68
AdaBoost	0.70	0.67	0.58	0.75
k-nearest neighbors	0.71	0.64	0.55	0.70
Random forest	0.71	0.70	0.55	0.81

Adding selected radiomics features to four clinical features (as described above) further improved the predictive performance. Among various machine learning models, SVM achieved the best performance with an AUC of 0.80 (Table 5).

**Table 5:** The performance of different machine learning models to predict the outcome using combination of clinical and radiomics features. AUC: area under the curve; SVM: support vector machine.

Model	AUC	Accuracy	Sensitivity	Specificity
AdaBoost	0.57	0.57	0.52	0.61
Decision Tree	0.62	0.55	0.55	0.54
k-nearest neighbors	0.69	0.66	0.52	0.77
Logistic Regression	0.72	0.65	0.61	0.68
Neural Network	0.72	0.69	0.67	0.70
Random Forest	0.75	0.69	0.55	0.79
SVM	0.80	0.76	0.67	0.84

## Discussion

iNPH is one of the most common differential diagnoses of neurodegenerative disorders and one of the few types of dementias that can be amenable to treatment if appropriately diagnosed and managed [1]. Diagnosis and management of the iNPH by no means are straightforward. iNPH has substantial overlap with other neurodegenerative and cognitive diseases in clinical presentations [6]. Especially, in elderly patients, “pure” iNPH is not always common. The exact physiopathology of iNPH is still controversial, but excessive CSF accumulation within the ventricular system eventually causes brain parenchymal damage and subsequent clinical symptoms. VP shunt placement remains the main treatment for these patients. Currently, after drainage of a large amount of CSF (tap test), gait improvement is the main technique utilized to select appropriate patients for shunt placement.

Same as the diagnosis, treatment response prediction after shunting in iNPH also remains challenging. This prediction was the subject of many studies with varying results. So far, evaluation of CSF pulsation patterns (CSF pressure changes secondary to cardiac pulsation) was not associated with treatment response [7,17-20]. Similar to CSF pulsation pattern, the patient’s age cannot predict the shunting response [7]. Based on the already existent guidelines, symptom improvement after “external lumbar drainage” is associated with a higher response rate after shunting, and this test is recommended for patient selection before shunting [7]. It seems even the combination of intracranial pressure monitoring and lumbar infusion is not able to predict the treatment response in iNPH after shunting [21]. It has been reported that abnormal single-photon emission computed tomography (SPECT) brain cerebral blood flow reactivity to acetazolamide can be associated with treatment response after shunting [7,22]. Besides, higher CSF velocity across the aqueduct has been reported to be associated with response to shunting [7,23-26]. However, other researchers did not observe such association, such as Dixon et al., who evaluated 49 patients who underwent shunting for iNPH. They concluded that CSF flow measurement at the level of the aqueduct could not predict the shunt response [23]. There is no significant association between the periventricular T2/FLAIR hyperintensities and treatment response to shunting [7,27]. In one study, small callosal angle, wide temporal horns, and occurrence of disproportionately enlarged subarachnoid space have been reported to be associated with treatment response [28], but this association was not detected in other similar studies [6,10,27].

Given the difficulty of treatment response prediction in iNPH, as mentioned above, we aimed to develop machine learning models to solve this challenging task. Moreover, we believe that MRI images contain information beyond the ability of human eyes. Subtle changes in the diameter of ventricles, their shape and sphericity, and subtle changes of CSF signal due to different intraventricular CSF motion may be able to stratify different patients and can help us separate out the responders from non-responders. In our study, by using the clinical data (iNPH grading scale and Modified Rankin Scale), we were able to predict the shunt response with moderate accuracy. As we predicted, adding selected predictive radiomics features improved the performance. Using the SVM, we predicted the response with an AUC of 0.8, which is acceptable for a challenging task and better than many prior studies. Interestingly, in one recently published study, the SVM had a better accuracy of iNPH diagnosis in comparison to radiologists [29]. In our study, the patients' age and sex were not predictive (neither in statistical analysis nor in machine learning models), which matches results from previous studies [6]. Our results match with another recently published study by Wu et al.; they evaluated 41 iNPH patients after shunt placement, after segmentation of 283 brain regions, volumetric analysis, and selection of the most predictive features, their machine learning model had high relation to the outcome (r = 0.80 for Tinetti scale and r = 0.88 for mini-mental state examination prediction) [30]. On the other hand, our segmentation was based only on ventricular segmentation and we believe it is more practical. In contrast to the lumbar tap test, our model is entirely based on non-invasive clinical and radiomics data, requires no additional test, and can eventually improve patient selection for iNPH shunting.

There are a few limitations in our conceptual study design. This study was a proof of concept- pilot study performed in a retrospective fashion. Non-structured medical data in medical records was extracted and converted to questionnaire format for model development which is not an optimum design for clinical data gathering. Although radiologists were blind to the patients’ outcomes, the physicians who did the post-op assessment knew that the patients have undergone shunt surgery. Additionally, only neurologic presentation was used for model development. Adding other clinical data, including cardiovascular risk factors as well as results of spinal tap test, may further improve the performance of the current predictive models. Besides, only the T2-weighted sequence was used for radiomics analysis for this study. Using multiparametric MRI sequences can potentially improve the performance of the final predictive models.

## Conclusions

NPH is one of the common etiologies of dementia. Accurate diagnosis of NPH is critical because it is potentially treatable. Ventricular shunting has been the standard of care of iNPH for a long time. However, shunting is not effective in all patients. Having an accurate estimate of the chance of treatment response for every patient will improve patient selection. The treatment response prediction in iNPH remains a challenging task. Many prior attempts failed to develop a standard prediction system with high accuracy. Given our findings, machine learning approaches based on the neurologic clinical manifestation of the patients are feasible for response prediction after ventricular shunt placement with acceptable performance (ROC of 0.71 in our model based on four clinical features: Cognitive impairment, Gait disturbance, Urinary incontinence, and Modified Rankin Scale). Importantly, adding the Radiomics analysis to clinical features can further augment the performance of such models (ROC of 0.8 in our model). We believe our Radiomics approach based on segmentation of the ventricular system on T2 is a simple approach that can be easily implemented in daily clinical practice without a financial challenge. Among different machine learning models, the random forest and support vector machine techniques are the most promising methods for treatment response prediction in iNPH. Further prospective studies are needed to evaluate the clinical implementation of such clinical/Radiomics machine learning approaches.
